# Lighting effects on optimal facial regions for remote heart rate measurement

**DOI:** 10.1038/s44325-026-00140-7

**Published:** 2026-06-23

**Authors:** Shuo Li, Mohamed Elgendi, Yanmin Zhu, Edmund Y. Lam, Carlo Menon

**Affiliations:** 1 Department of Electrical and Computer Engineering, The University of Hong Kong, Pokfulam, Hong Kong China; 2 Biomedical and Mobile Health Technology Lab, ETH Zürich, Zürich, Switzerland; 3 Department of Information Technology and Electrical Engineering, ETH Zürich, Zürich, Switzerland; 4 Department of Biomedical Engineering and Biotechnology, Khalifa University of Science and Technology, Abu Dhabi, UAE; 5 Healthcare Engineering Innovation Group (HEIG), Khalifa University of Science and Technology, Abu Dhabi, UAE

**Keywords:** Cardiology, Diseases

## Abstract

Remote photoplethysmography (rPPG) can evaluate real-time changes in blood flow volume by capturing facial videos and analyzing the color changes. Although the rPPG technique enables contactless heart rate (HR) monitoring, it remains highly susceptible to ambient lighting variations. Previous studies have demonstrated the critical influence of facial skin detection on the extracted rPPG signals. In this research, we defined 15 facial regions of interest (ROIs) based on anatomical criteria and evaluated their HR measurement performance on two public datasets (BUAA-MIHR and MMPD) using four representative rPPG algorithms (CHROM, LGI, OMIT, and POS). The experimental results confirmed that the glabella, nasal dorsum, and malar regions consistently serve as robust physiological signal sources under complex illumination environments. Furthermore, we revealed the significant advantage of multi-ROI combinations compared to both single-ROI and holistic-face strategies. Our work provides data-supported insights for establishing novel HR measurement pipelines with enhanced accuracy and robustness.

## Introduction

Cardiac function is one of the most crucial indicators to reflect the state of human health. Heart rate (HR) generally serves as the most straightforward physiological index, which can objectively reflect the cardiovascular condition. With the growing public awareness of personal health management, regular physical examinations can no longer satisfy the demands for real-time and cost-effective health monitoring^1,2^. In this context, traditional contact-based HR measurement techniques—such as electrocardiography (ECG)^3^ and contact-based photoplethysmography (cPPG)^4,5^—while widely regarded as the gold standard for clinical settings or consumer-grade devices, are typically unsuitable for general measurement scenarios in the absence of specialized equipment^6,7^. The primary limitations are the physical discomfort caused by the direct attachment of electrodes and sensors to the skin, as well as the restricted subject mobility resulting from such methods^8,9^.

In recent years, remote photoplethysmography (rPPG) has emerged as a representative alternative solution^10^. It uses facial video sequences captured by ordinary cameras as raw input data to analyze subtle color changes in human skin pixels induced by temporal blood volume variations. The typical advantages of this technology lie in its on-site monitoring capability and the easy accessibility of measurement equipment. Simply extracting the time-series signal of the green (G) channel is generally considered as the earliest rPPG method^11^, yet the quality of the acquired signal is often inconsistent. Subsequent algorithms integrated information from the red (R) and blue (B) channels to enhance signal robustness. In the quantitative analyses conducted by Haugg et al.^12^, physical model-driven algorithms represented by plane-orthogonal-to-skin (POS)^13^ and the chrominance-based method (CHROM)^14^, as well as data-adaptive projection algorithms represented by orthogonal matrix image transformation (OMIT)^15^ and local group invariance (LGI)^16^, exhibited superior performance compared with blind source separation algorithms such as independent component analysis (ICA)^17^ and principal component analysis (PCA)^18^, thereby yielding HR measurement results with lower error. While early rPPG research focused predominantly on HR estimation, recent studies have extended its application to other clinically relevant parameters, including blood pressure^19^ and blood oxygen saturation (SpO_2_)^20^.

However, since rPPG algorithms inherently rely on detecting variations in light reflection from the skin surface, the obtained physiological signals are highly susceptible to noise interference from various sources in the measurement environment. Multiple studies have shown that motion-induced artifacts can significantly affect the HR measurement accuracy^21–23^. Other latent factors, such as ambient lighting distributions^24^, subject skin colors^25^, and video compression methods^26,27^, also significantly influence the measurement performance. To address low-light challenges specifically, recent efforts have explored hardware-level exposure optimization^28^, physics-based image enhancement^29^, and learning-based illumination adaptation^30^. Furthermore, the importance of identifying appropriate facial regions of interest (ROIs) has been highlighted in the research of Pirnar et al.^31^, particularly in challenging scenarios. Kim et al.^32^ systematically evaluated the quality of rPPG signals extracted from different facial ROIs under ideal measurement environments. The results indicated that skin thickness and ROI size might be potential affecting factors. Although several studies have confirmed that the forehead and cheek regions typically yield higher-quality rPPG signals^33,34^, the glabella has been identified as the optimal ROI during motion and cognitive tasks compared to other anatomically defined regions^35^. Other researchers have designed different types of signal quality indices (SQIs) to dynamically select target ROIs that yield high-fidelity physiological signals^36,37^.

In real-world applications associated with distinct measurement scenarios, variations in illumination intensity and light source types are regarded as critical bottlenecks affecting rPPG signal quality. The human face exhibits a complex anatomical topography, characterized by dermal and epidermal skin thickness, non-uniform surface orientations, and an intricate distribution of underlying vascular beds^38^. Therefore, changes in illumination conditions may cause uneven influence on the physiological signals extracted from different facial regions. Nevertheless, quantitative comparison of facial ROI performance under diverse lighting conditions has rarely been explored. In this research, we provide specific definitions of 15 human facial ROIs based on keypoints extracted using MediaPipe Face Mesh^39^, a real-time deep learning-based facial landmark detection framework. We define the facial regions with the consideration of both anatomical structure and ROI area balance. Two publicly available datasets are involved in the quantitative analyses: BUAA-MIHR^29^ and MMPD^40^. To ensure the experimental comprehensiveness, we compare the HR measurement accuracy for individual ROIs, combination of multiple ROIs, and the holistic face. The research outcomes provide substantial data-supported insights with coverage of common lighting scenarios, laying the foundation for developing more robust and widely applicable rPPG-based HR measurement systems in complex real-world situations. Unlike prior ROI-based studies, this work provides the first systematic cross-dataset and cross-illumination evaluation integrating anatomical ROI design, statistical validation, and multi-ROI fusion analysis.

## Results

**Fig. 1:**
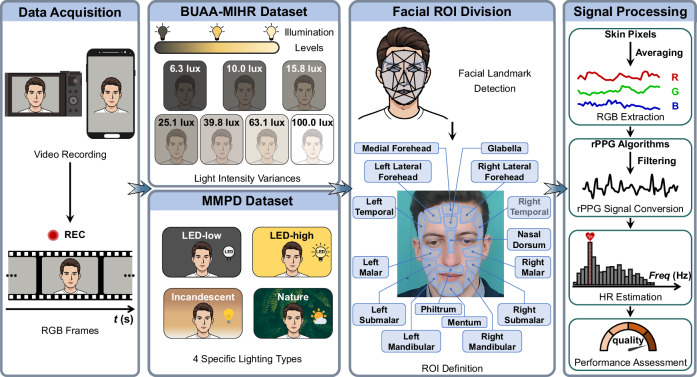
Schematic workflow of the proposed heart rate (HR) monitoring pipeline and region of interest (ROI) performance evaluation based on remote photoplethysmography (rPPG). Systematic quantitative experiments were conducted on two public datasets: BUAA-MIHR^29^ (featuring indoor LED illumination variances) and MMPD^40^ (including four specific lighting types: low LED, high LED, incandescent, and natural light). The human facial region was partitioned into 15 anatomically-defined ROIs for further performance assessment. Open-licensed icons were adapted from SVG Repo (https://www.svgrepo.com) and under the Creative Commons Zero v1.0 Universal License (https://creativecommons.org/publicdomain/zero/1.0/). The human face image was sourced from the UBFC-rPPG dataset (subject 47, who has consented to present this image in publications). Dataset URL: https://sites.google.com/view/ybenezeth/ubfcrppg/.

**Fig. 2:**
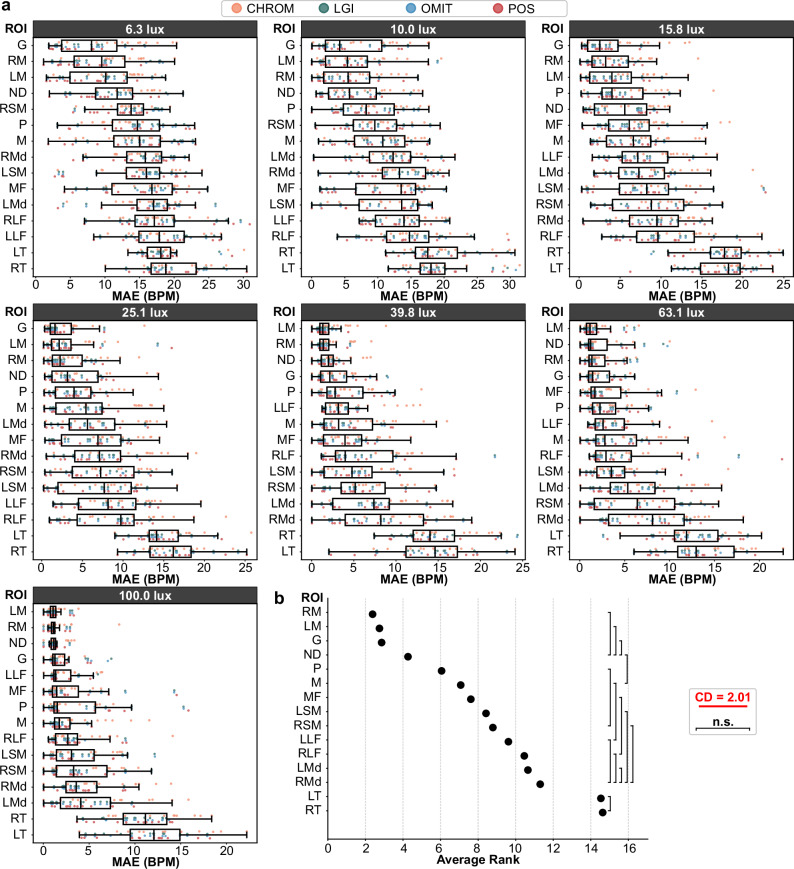
Heart rate (HR) measurement accuracy assessment of different facial regions of interest (ROIs) on the BUAA-MIHR dataset^29^. **a** Box plots of mean absolute error (MAE) performance in different illumination levels: 6.3, 10.0, 15.8, 25.1, 39.8, 63.1, and 100.0 lux. For each ROI, the HR measurement performance of four rPPG algorithms (CHROM^14^, LGI^16^, OMIT^15^, and POS^13^) is visualized using distinct colors accordingly. The lines within the boxes represent the median values. **b** Critical difference (CD) diagram. Facial ROIs are ordered from top (best) to bottom (worst) based on their average MAE ranks across all illumination levels. Black bars denote groups of ROIs with non-significant (n.s.) differences in MAE at *α* = 0.05 (Nemenyi post-hoc test). The red bar corresponds to the threshold for statistical significance.

To systematically quantify the performance variations of single facial ROIs in rPPG-based HR measurement under distinct ambient lighting conditions, we first evaluated the measurement errors of 15 anatomically defined facial ROIs across seven LED illumination intensity levels (6.3 to 100.0 lux) based on the BUAA-MIHR dataset. As shown in Fig. 2, for all four representative rPPG algorithms (CHROM, LGI, OMIT, and POS), the HR measurement performance of all facial ROIs generally improved with the gradually increasing lighting intensity. Under extremely dim environments (6.3 lux), only the glabella and right malar achieved median mean absolute errors (MAEs) below 10 beats per minute (BPM). When the illumination intensity was elevated to 15.8 lux or above, the median MAEs for all regions—except the left and right temporal—fell below 10 BPM. Among them, the measurement accuracy of the glabella, left malar, right malar, nasal dorsum, and philtrum stabilized below 5 BPM in environments of 25.1 lux and higher. Notably, the top-4 regions (the glabella, left malar, right malar, nasal dorsum) consistently maintained MAEs within 5 BPM for all measurement results. Figure 2b presents the critical difference (CD) diagram with the results of average ranks and Nemenyi post-hoc testing to statistically validate the significant differences among different ROIs. According to the diagram, the right malar, left malar, glabella, and nasal dorsum significantly outperformed other regions, with no significant internal differences. In comparison, the forehead region (comprising the medial, left lateral, and right lateral forehead defined in Fig. 1), which is normally regarded as an excellent ROI candidate, yielded only mediocre performance. Furthermore, the submalar regions performed significantly worse than the malar regions, indicating inherent variations in physiological signal quality even within the commonly used cheek region.

Apart from variations in illumination intensity, different light source types can also exert diverse levels of impact on the rPPG signal extraction process. In the MMPD dataset, we evaluated the performance of different facial ROIs under four illumination conditions (LED-low, LED-high, incandescent, and nature) involving subject motions (stationary, rotation, talking, and walking). The results show that changes of light source types also exert a noticeable influence on the HR measurement accuracy of different facial ROIs. Consistent with the results on the BUAA-MIHR dataset, the glabella, left malar, right malar, nasal dorsum, and philtrum still secured the highest average ranks among all ROIs, with the top-4 regions maintaining a statistically significant advantage over the others. Conversely, the nasal dorsum and glabella significantly outperformed the left malar and right malar in the MMPD dataset. This result shows that under broadband light sources such as incandescent lamps and natural light, regions along the facial midline can maintain higher signal fidelity.

To determine whether the observed HR measurement performance rankings of individual ROIs were influenced by specific signal extraction mechanisms of the evaluated algorithms, we assessed the consistency of ROI rankings across the four rPPG methods using Kendall’s coefficient of concordance (*W*). As summarized in Table 1, the statistical tests yielded exceptionally high concordance coefficients (*W*≥0.864) across all LED illumination intensities in the BUAA-MIHR dataset and all lighting types in the MMPD dataset. Moreover, all statistical tests demonstrated high significance (*p* < 0.001). These quantitative results indicate a strong, statistically significant consensus among the four structurally diverse algorithms (CHROM, LGI, OMIT, and POS) regarding the relative performance of the 15 facial ROIs under complex lighting conditions.

**Table 1:** Kendall’s coefficient of concordance for ROI rankings across four rPPG algorithms

Dataset	Illumination Condition	Kendall’s *W*	*p*-value
BUAA-MIHR^29^	6.3 lux	0.864	1.1393 × 10^−5^
	10.0 lux	0.916	3.7283 × 10^−6^
	15.8 lux	0.982	8.6579 × 10^−7^
	25.1 lux	0.951	1.7047 × 10^−6^
	39.8 lux	0.960	1.4035 × 10^−6^
	63.1 lux	0.967	1.1965 × 10^−6^
	100.0 lux	0.956	1.5302 × 10^−6^
MMPD^40^	LED-low	0.965	1.2596 × 10^−6^
	LED-high	0.931	2.6783 × 10^−6^
	Incandescent	0.957	1.5183 × 10^−6^
	Nature	0.905	4.7504 × 10^−6^

**Fig. 3:**
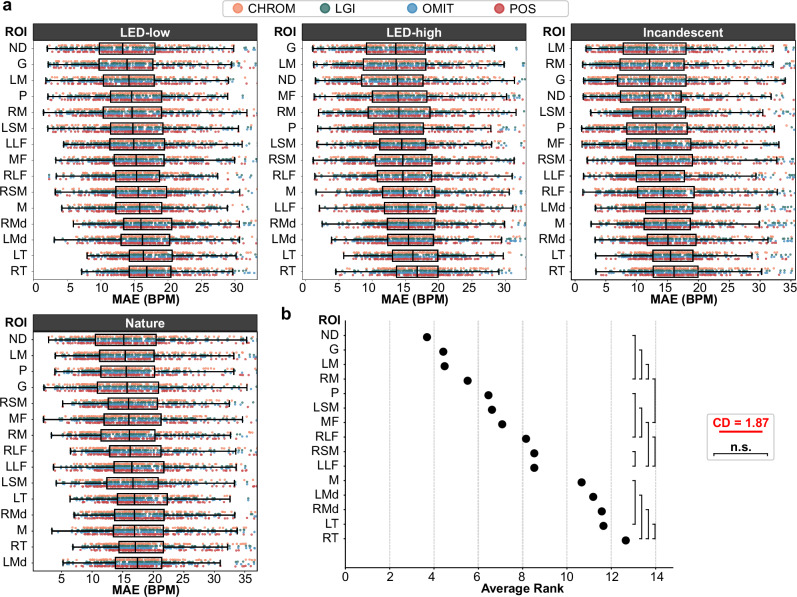
Heart rate (HR) measurement accuracy assessment of different facial regions of interest (ROIs) on the MMPD dataset^40^. **a** Box plots of mean absolute error (MAE) performance in four specific lighting conditions: LED-low, LED-high, incandescent, and nature. For each ROI, the HR measurement performance of four rPPG algorithms (CHROM^14^, LGI^16^, OMIT^15^, and POS^13^) is visualized using distinct colors accordingly. The lines within the boxes represent the median values. **b** Critical difference (CD) diagram. Facial ROIs are ordered from top (best) to bottom (worst) based on their average MAE ranks across all illumination levels. Black bars denote groups of ROIs with non-significant (n.s.) differences in MAE at *α* = 0.05 (Nemenyi post-hoc test). The red bar indicates the CD value, which serves as the threshold for statistical significance.

The statistical tests evaluated the agreement among CHROM^14^, POS^13^, OMIT^15^, and LGI^16^ regarding the HR measurement performance rankings of 15 facial ROIs on the BUAA-MIHR dataset (Fig. 2) and MMPD dataset (Fig. 3), respectively. A value of *W* closer to 1 indicates stronger agreement.

**Fig. 4:**
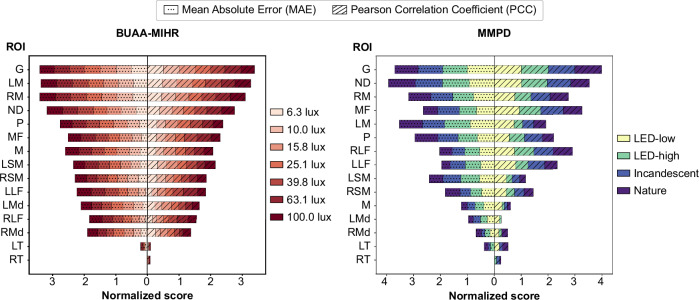
Normalized score evaluation of individual facial regions of interest (ROIs). The two-sided stacked bar charts are utilized to present normalized scores derived from the Pearson correlation coefficient (PCC) and mean absolute error (MAE). Different colors within the stacked bars denote specific illumination levels (from 6.3 to 100.0 lux) and lighting conditions (LED-low, LED-high, incandescent, and nature). The ROIs are sorted in descending order from top to bottom by the total sum of normalized scores.

In order to provide a more comprehensive performance comparison, we applied the Pearson correlation coefficient (PCC) to evaluate the waveform morphological quality of rPPG signals. By combining both MAE and PCC via min-max normalization (Equations (1) and (2)), we computed the corresponding normalized scores for each ROI. As shown in the two-sided stacked bar charts in Fig. 4, it can be observed that the glabella achieved the highest overall scores in both datasets. This discovery is highly consistent with a previous study incorporating common physical and cognitive tasks^35^, demonstrating the stability and universal applicability of this region across various complex environments. Overall, six regions—the glabella, left malar, right malar, nasal dorsum, medial forehead, and philtrum—obtained the highest normalized score rankings across both datasets. In the BUAA-MIHR dataset, the glabella, left malar, right malar, and nasal dorsum dominated the top scores, presenting a high degree of consistency across all illumination levels from 6.3 to 100.0 lux. In the MMPD dataset, which contains incandescent and natural light together with more subject activities, the nasal dorsum outperformed the left and right malar. By contrast, the perioral and lower facial regions (e.g., mentum and mandibular regions) achieved relatively low comprehensive scores. These results collectively suggest that regions in the upper middle of the face and rich in superficial vascular beds not only provide accurate HR estimates, but also sustain high-fidelity pulse waveform patterns in varying lighting environments.

**Fig. 5:**
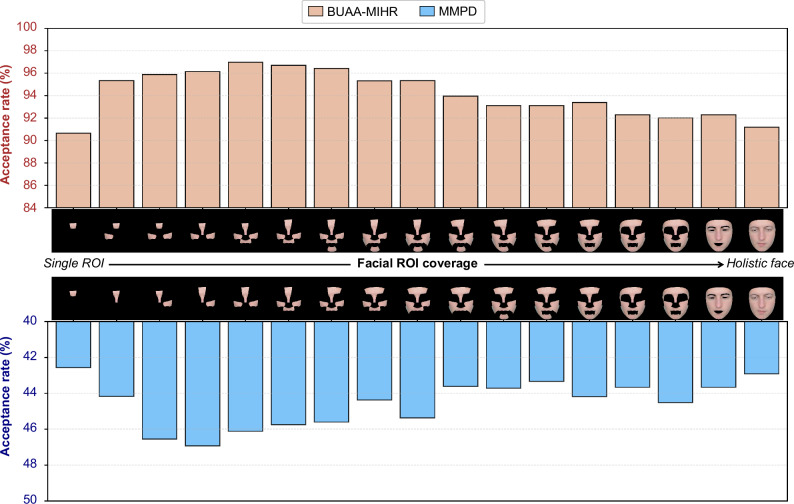
Comparison of heart rate (HR) measurement acceptance rates with gradual expansion of facial regions of interest (ROIs) based on remote photoplethysmography (rPPG). The expansion of facial ROIs follows the ranking in Fig. 4. The two-sided bar chart illustrates the acceptance rates of HR measurement assessed on the BUAA-MIHR dataset^29^ (top) and the MMPD dataset^40^ (bottom). The horizontal axis presents the progressive expansion of facial ROI coverage. The second-to-last image represents the holistic face with only skin pixels (excluding eyebrows, eyes, and mouth), while the rightmost image represents the unmasked holistic face. The corresponding Bland--Altman analyses are provided in Supplementary Information (Note 1). The human face image was sourced from the UBFC-rPPG dataset (subject 47, who has consented to present this image in publications). Dataset URL: https://sites.google.com/view/ybenezeth/ubfcrppg/.

**Fig. 6:**
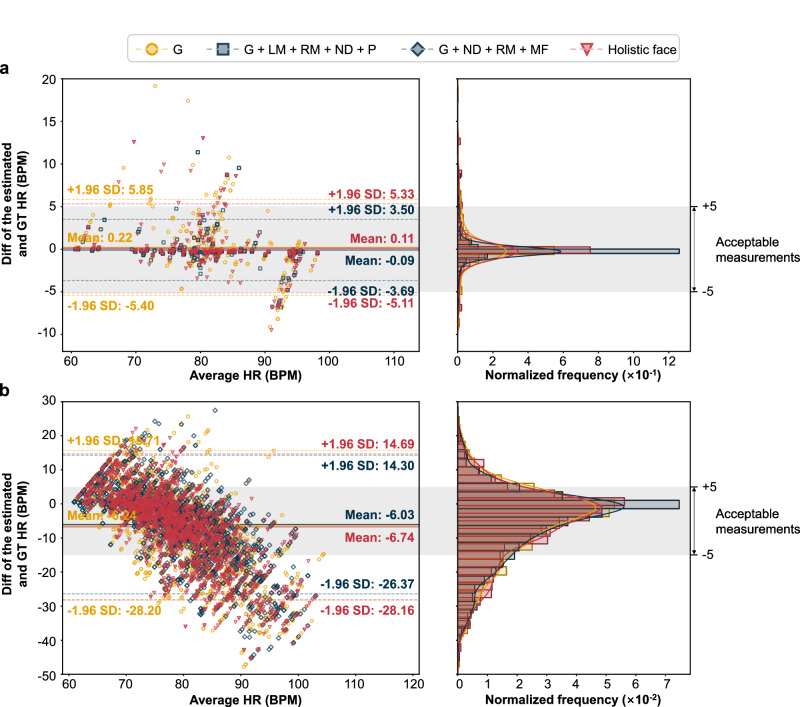
Bland–Altman analysis and frequency histogram of heart rate (HR) measurement accuracy of glabella, the best-performing ROI combination, and holistic face. **a** BUAA-MIHR^29^. **b** MMPD^40^. Each data point in the Bland--Altman plots (left side) corresponds to a pair of measurements between the estimated HR versus the ground truth (GT) within 60 s. The acceptable range of HR measurement is set as MAE≤5BPM based on the Consumer Technology Association (CTA) standards^41,42^.

Beyond individual facial regions, we further investigated the synergistic effects of combining multiple high-performing ROIs compared to single-region or holistic-face extraction. Figure 5 illustrates the trend of HR measurement acceptance rate (defined as MAE≤5BPM for a 1-min video sequence, based on the Consumer Technology Association (CTA) standards^41,42^) as the facial ROI coverage gradually expands according to the rankings of normalized scores in Fig. 4. The details of measurement results are shown in the form of Bland–Altman plots in Supplementary Information (Note 1). The results on the BUAA-MIHR dataset show that using the best-scoring single ROI (glabella) alone yielded an acceptance rate slightly above 90%. As the ROI coverage expanded, the acceptance rate reached the peak (around 97%) when the ROI combination included the top-5 regions. As lower-ranking regions were subsequently incorporated into the combination until extending to the holistic face, the acceptance rate gradually declined. This upward-then-downward trend was similarly validated in the MMPD dataset: the combination of the top-4 regions achieved the highest HR measurement acceptance rate (approximately 47%), whereas both the single optimal ROI (glabella) and the holistic-face strategies fell below 43%. The Bland–Altman analysis and the corresponding frequency histograms of measurement errors in Fig. 6 provide further quantitative support for the advantage of this multi-region combination. In the BUAA-MIHR dataset (Fig. 6a), the 95% limits of agreement (LoA) for the single optimal ROI (glabella) and the holistic face were [−5.40, 5.85] BPM and [−5.11, 5.33] BPM, respectively. By contrast, the LoA for the top-5 ROI combination significantly narrowed down to [−3.69, 3.50] BPM, with a minimum mean bias of −0.09 BPM. Similar results were observed in the MMPD dataset (Fig. 6b), where the top-4 ROI combination produced a tighter error distribution range than both the glabella and the holistic face. The frequency histograms of errors on the right side clearly illustrate that, selectively fusing the best-performing individual ROIs concentrates a larger proportion of measurements within the acceptable ± 5 BPM range. These data-supported findings demonstrate that utilizing the holistic face for spatial averaging is susceptible to noise interference; conversely, selectively combining a few noise-robust regions is the optimal strategy to enhance rPPG-based HR measurement performance under complex lighting conditions.

## Discussion

Multiple previous studies have emphasized the importance of selecting appropriate skin regions in rPPG-based measurement systems, particularly in complex application scenarios. During the process of translation from laboratory settings to practical applications, the diversity of ambient illumination conditions remains one of the factors influencing measurement precision. For example, natural lighting generally holds a dominant proportion in outdoor environments, whereas the spectral characteristics of incandescent lamps and LED lights under indoor conditions can exert diverse degrees of influence on the signal extraction process. Furthermore, environmental changes are often accompanied by fluctuations in light intensity, such as low-light conditions during nocturnal vital sign monitoring, nighttime driving, and overnight security, or higher-illumination settings required for telemedicine, indoor fitness activities, and remote working. Our study systematically quantified the rPPG-based HR measurement performance of different anatomically defined facial ROIs across multiple levels of illumination intensity (6.3 to 100.0 lux) and different light source types (LED, incandescent, and natural light). The quantitative experimental results demonstrate significant disparities in the HR measurement performance of different facial regions under varying lighting conditions. In the comparative analysis of individual ROIs presented in Figs. 2, 3, the glabella, nasal dorsum, and malar regions consistently achieved the lowest median MAE, showing statistically significant advantages over the remaining 11 regions. Moreover, for conditions involving various light sources and subject motions, the ROIs along the facial midline (glabella and nasal dorsum) significantly outperformed the malar regions. The results in Fig. 4 illustrate that the signal quality extracted from the glabella presented the highest stability across both datasets. This finding is remarkably consistent with a previous study on physical activities and cognitive tasks^35^, further proving the glabella as the premier choice in various challenging measurement scenarios. In essence, the rPPG technique is the process of capturing the diffuse reflectance of light penetrating the skin and being modulated by the dermal microvascular network. According to a recent study in high-frequency ultrasonography and dermatology, the dermal layers of the glabella and nasal dorsum are relatively thin with minimal subcutaneous fat^43^. This anatomical characteristic reduces the amount of incident light that reaches non-pulsatile tissue layers, thereby diminishing unnecessary scattering and absorption losses. Thus, even under very dim illumination (e.g., 6.3 lux), the diffuse reflection light reaching the camera sensor still contains sufficient physiological information. Moreover, angiographic and immunofluorescence studies have also shown that the forehead, glabella, and malar regions have a high concentration of superficial capillary networks and subdermal vascular plexuses^44,45^. This anatomical structure might be one of the key principles that support their ability to serve as high-fidelity pulse signal sources.

Another important finding of this article is that when compared to the traditional protocols of either extracting signals from a single part of the face or from the holistic face, a proper combination of several best-performing regions may result in the highest acceptance rate of HR measurement that is illustrated in Figs. 5, 6. This non-linear, increasing, then decreasing acceptance rate pattern as the ROI coverage area is increased indicates that there must be a trade-off: when gradually expanding the target area to eliminate quantization noise of camera sensors and localized temporal illumination artifacts, further increases in ROI coverage area inevitably include regions that contain less pulsatile information. Inclusion of such regions can have severe effects on the quality of the rPPG signal. The quantitative data illustrated by the Bland–Altman analysis further indicate that the 95% LoA of the optimal ROI combination lies within a smaller range, in comparison with the single-ROI as well as the holistic-face strategies that generated more measurement outliers. Recent literature has been investigating various types of SQIs to dynamically estimate rPPG signal fidelity for ROI selection. The importance of integrating SQIs into the current rPPG measurement pipelines is supported by our experimental results.

It should be mentioned that the statistical findings of Kendall’s W indicate that the ranking of individual ROIs across four representative rPPG algorithms, including physical model-driven algorithms (POS and CHROM) and data-adaptive projection algorithms (OMIT and LGI), is very consistent. Accordingly, the high signal fidelity of individual ROIs such as the glabella, nasal dorsum, or malar regions is not induced by algorithmic bias but rather determined by their inherent anatomical and optical characteristics. Practically, this finding implies that future studies in the field of rPPG technology do not have to necessarily involve developing learning-based signal conversion models, which are increasingly complex. The lightweight algorithms are also capable of attaining consumer-grade accuracy rates, even in challenging scenarios, by focusing on robust signal sources on the face.

Finally, we note that improving rPPG measurement robustness under low-light conditions is an active research area, with recent advances including hardware optimization (e.g., adaptive exposure control^28^) and image enhancement techniques^29,30^. Our work offers a complementary spatial strategy: by selecting and combining anatomically robust facial ROIs, we achieve competitive HR measurement accuracy using standard cameras and lightweight algorithms, without requiring specialized hardware settings or learning-based enhancement.

Although this study conducted quantitative analyses on two representative datasets encompassing diverse lighting conditions, there is still room for further exploration. First, future research should concentrate more on addressing the algorithmic performance barriers of dark-skinned subjects through customized spectral feature extraction strategies. Second, since real-world application scenarios (e.g., in-vehicle monitoring or nighttime security) are often associated with directional and transient lighting changes, future work should focus on developing a spatiotemporal adaptive ROI fusion framework to automatically shift the weighting toward regions with more physiological signals. In parallel, integrating temporal HR-level correction based on cardiac dynamics into such a fusion framework could provide a more robust solution for dynamic lighting and motion conditions^46^. Additionally, the evaluations in this study primarily focused on the visible light spectrum. In zero-illuminance scenarios, such as clinical sleep monitoring, near-infrared (NIR) imaging is a typical alternative approach. Given the significant differences in hemoglobin absorption coefficients and scattering characteristics between NIR and visible light, it remains necessary to validate the applicability of the identified optimal facial regions under the NIR spectrum.

## Methods

### Datasets

In this research, our experiments utilize two publicly available datasets: BUAA-MIHR and MMPD. While both datasets provide facial videos and the corresponding ground truth cPPG data under varied illumination conditions, their experimental emphases differ. BUAA-MIHR contains facial video recordings under multiple levels of LED light intensities, while the data collection of MMPD was more focused on different types of light sources and physical activities.

#### BUAA-MIHR

The BUAA-MIHR dataset became available first together with an image enhancement algorithm developed by Xi et al.^29^. It was built to assess the performance of rPPG-based HR measurement in low-light and multi-level illumination conditions. There are 15 healthy subjects in the dataset, between the ages of 18–30 years (12 males and 3 females). The facial videos were recorded using a Logitech HD Pro C930E webcam with a resolution of 640 × 480 pixels, a frame rate of 30 fps, and a single video duration of 60 s. The recording was done entirely in a darkroom, and an adjustable LED lamp (Panasonic HHLT0623) was used to mimic the effect of the interior lighting conditions at various brightness levels. A professional illuminometer was used to measure the face illumination (BENETECH GM1030), while the ground truth cPPG signals were acquired with a fingertip pulse oximeter (CONTEC CMS50E). As shown in Fig. 1, our experimental analyses incorporated illuminance levels of 6.3, 10.0, 15.8, 25.1, 39.8, 63.1, and 100.0 lux.

#### MMPD

The MMPD dataset was designed and proposed to simulate challenging real-world measurement scenarios^40^. This dataset encompasses a wide range of data collected in different perspectives, including skin tone, light source type, and physical activity. Unlike the BUAA-MIHR dataset, which primarily includes East Asian skin tones, MMPD covers Fitzpatrick skin types III to VI, thereby providing representation for subjects with dark skins. As shown in Fig. 1, this dataset is comprised of four lighting conditions: low-light LED, high-light LED, incandescent lamp, and natural light. Furthermore, it includes three types of movements—head rotation, talking, and walking—together with recording data from stationary subjects. In the experiments, we utilized data across these four motion tasks under four lighting conditions. Facial videos of all 33 subjects were captured using the front camera of a smartphone (Samsung Galaxy S22 Ultra) with a resolution of 1280 × 720, a frame rate of 30 fps, and a duration of 60 s per video. The cPPG signals acquired by the pulse oximeter (HKG-07C+) serve as the synchronous waveform ground truth.

### ROI division of human face

Precise frame-by-frame localization and segmentation of facial ROIs are fundamental to maintaining the rPPG signal quality. Many previous methods rely on bounding box detection for subsequent signal processing steps^47^, while other studies focused on detecting specific regions with richer pulsatile signals (e.g., cheeks and the entire forehead)^34,48,49^. Although recent studies have compared the performance of different single facial ROIs under ideal lighting conditions in cognitive or physical tasks^32,35^, the impact of illumination changes in real-world measurement scenarios remains insufficiently explored. Furthermore, quantitative analyses of the trade-off between extracting signals from individual regions versus the holistic face are currently lacking: small ROIs are extremely vulnerable to spatial quantization noise (especially in low-light conditions), while overly large ROIs are prone to non-uniform reflections or partial occlusions that can obscure physiological signals.

Research conducted in the field of dermatology and high-frequency ultrasonography has discovered that topographical variations of human facial skin are highly heterogeneous, including the topographical variations of dermal thickness, subcutaneous tissue depth, and the density of underlying capillary beds^43–45^. In principle, rPPG techniques are based on detecting the diffuse reflected light—that is, the light that penetrates the skin, interacts with the superficial vascular tissue, and then exits—with the anatomical differences directly affecting the effective optical path length. Different regions of the face exhibit dissimilar absorption and reflection properties under various ambient lighting conditions, and hence influence the resulting signal quality.

We present a structured ROI division strategy to analyze performance variations across facial regions under different lighting conditions; an overview of facial ROI division is provided in the workflow diagram (Fig. 1). The proposed division strategy considers the facial anatomical consistency, but ensures the area balance across the defined facial regions, minimizing the baseline noise variances in the spatial averaging process. In particular, we utilized the 468-point facial landmarks by MediaPipe Face Mesh^39^ that allowed us to precisely divide target regions. As shown in Table 2, the holistic face is segmented into 15 independent ROIs, including the medial forehead, glabella, nasal dorsum, and other regions. Each ROI is represented by a closed polygon using a sequence of keypoint indices. This topology-based definition guarantees that the target ROI is able to fit the corresponding anatomical subunits across various subjects, head postures, and varying lighting conditions.

**Table 2:** Keypoint list of proposed facial ROIs

ROI name	Abbreviation	Keypoint list
medial forehead	MF	10, 109, 108, 151, 337, 338
glabella	G	151, 108, 107, 55, 8, 285, 336, 337
left lateral forehead	LLF	67, 103, 104, 105, 66, 107, 108, 109
right lateral forehead	RLF	297, 338, 337, 336, 296, 334, 333, 332
left temporal	LT	103, 54, 21, 162, 127, 116, 143, 156, 70, 63, 105, 104
right temporal	RT	332, 333, 334, 293, 300, 383, 372, 345, 356, 389, 251, 284
nasal dorsum	ND	8, 55, 193, 122, 196, 3, 195, 248, 419, 351, 417, 285
left malar	LM	126, 100, 118, 117, 116, 123, 147, 187, 205, 203, 129, 209
right malar	RM	355, 429, 358, 423, 425, 411, 376, 352, 345, 346, 347, 329
philtrum	P	2, 97, 98, 203, 186, 185, 40, 39, 37, 0, 267, 269, 270, 409, 410, 423, 327, 326
left submalar	LSM	203, 205, 187, 147, 177, 215, 138, 172, 136, 135, 212, 186
right submalar	RSM	423, 410, 432, 364, 365, 397, 367, 435, 401, 376, 411, 425
left mandibular	LMd	186, 43, 204, 194, 32, 140, 176, 149, 150, 136, 135, 212
right mandibular	RMd	410, 273, 424, 418, 262, 369, 400, 378, 379, 365, 364, 432
mentum	M	18, 83, 182, 194, 32, 140, 176, 148, 152, 377, 400, 369, 262, 418, 406, 313

The indices of facial keypoints can be referenced from MediaPipe Face Mesh. These keypoints are used to define a specific region of human face.

### Remote heart rate measurement pipeline

The signal processing workflow from raw video input to HR estimation is illustrated in Fig. 1. For each consecutive video frame, we extracted 468 two-dimensional facial keypoints using MediaPipe Face Mesh^39^. Based on the list of keypoints defined in Table 2, we precisely identified the target ROIs, including regions such as the glabella, medial forehead, and nasal dorsum. For each ROI per frame, the average pixel intensities across RGB channels were calculated to generate the raw RGB time-series traces.

In order to ensure a comprehensive assessment, we utilized four representative rPPG algorithms to recover the blood volume pulse variations from the raw RGB traces: CHROM^14^, POS^13^, OMIT^15^, and LGI^16^. As typical physical model-driven algorithms, both CHROM and POS rely on the dichromatic reflection model to differentiate diffuse and specular reflection, although the two algorithms have different emphases. The CHROM algorithm works on the standardized skin-tone assumption, mapping normalized RGB traces onto a chrominance subspace, and then applying an adaptive *α*-tuning mechanism to reduce specular noise. To address the shortcomings of the fixed skin-tone assumption under non-white illumination, the POS approach dynamically determines a projection plane perpendicular to the temporally normalized skin-tone vector. The in-phase pulsation component is extracted in the projected signal. Data-adaptive projection algorithms like OMIT and LGI, on the other hand, are based on the statistical characteristics of the input data instead of pre-defined physical models. The OMIT method utilizes a thin QR decomposition of the RGB matrix. OMIT projects the data onto an orthogonal subspace by capturing the dominating motion and compression noise on the first column of the resulting orthogonal matrix *Q*. The LGI algorithm deals with these motions by considering the rigid and non-rigid motions of the face as perturbation transformations of the Lie group. It uses orthogonal projection to map the RGB features into a complementary null space to produce an extracted signal that is insensitive to motion and illumination interference. The conversion from RGB to rPPG was implemented based on the pyVHR framework^50^.

To further enhance the signal quality, we employed a post-filtering stage to eliminate non-pulsatile frequency components. Specifically, the original rPPG signal was processed using a sixth-order Butterworth bandpass filter with cut-off frequencies of 0.65 Hz and 4.0 Hz, corresponding to a normal HR range of 39 to 240 BPM. This filter configuration follows established practice in pyVHR. The comparative analyses of alternative filters (the 4th-order Chebyshev Type II and 2nd-order Butterworth) are provided in Supplementary Information (Note 2), confirming that our main findings are robust to reasonable variations in filter choice^51^. A sliding window operation was implemented with a window length of 6 s and a step size of 1 s. For each windowed signal segment, the power spectral density (PSD) of the resulting signal was calculated using Welch’s method^52^, and the final estimated HR was obtained by identifying the frequency that corresponds to the dominant spectral peak within the valid frequency band.

### Evaluation metrics

Overall, the comprehensive performance of rPPG-based HR estimation can be evaluated from two perspectives: HR measurement accuracy and waveform-level quality. In this study, we utilized two widely recognized statistical metrics: MAE and PCC.

MAE is the most direct and broadly used evaluation metric in HR measurement. It is applied to evaluate the absolute accuracy of HR estimation relative to the reference data, directly measuring the average estimation errors in BPM. The definition of the MAE metric is as follows:

1 $$\mathrm{MAE}=\frac{1}{N}\mathop{\sum }\limits_{n=1}^{N}\left|H{R}_{\mathrm{est}}(n)-H{R}_{\mathrm{gt}}(n)\right|,$$

where *N* denotes the total number of valid HR measurement windows, while *HR*_est_(*n*) and *HR*_gt_(*n*) represent the estimated and ground truth HR for the *n*-th window, respectively.

While MAE can reflect the absolute error of HR estimation, it fails to capture the consistency of temporal trends and the waveform-level quality. Thus, we adopted PCC, which is calculated directly from time-series signals rather than from the final HR values. This metric measures the linear consistency between the extracted rPPG signal and the reference cPPG signal, reflecting the ability to restore the pulse waveform morphology. The computing formula of PCC is as follows:

2 $$\rho =\frac{\,\mathrm{cov}\,({S}_{{\rm{rPPG}}},{S}_{{\rm{cPPG}}})}{{\sigma }_{{S}_{{\rm{rPPG}}}}{\sigma }_{{S}_{{\rm{cPPG}}}}},$$

where *S*_rPPG_ and *S*_cPPG_ represent the acquired rPPG signal and the corresponding ground truth cPPG signal, respectively; cov( ⋅ ) denotes the covariance, and *σ* represents the standard deviation. This metric ranges from -1 to 1.

To integrate these two metrics and provide a comprehensive visual representation across different illumination conditions, we applied a normalization process in visualizing Fig. 4. Since MAE and PCC have opposite optimization directions and different numerical ranges—where a lower MAE indicates better HR measurement accuracy, whereas a higher PCC represents superior waveform quality—we employed min-max normalization to scale both metrics within the range of [0, 1]. This normalization procedure enables seamless integration of both metrics across all experimental conditions to calculate a comprehensive score that reflects the overall performance of each ROI.

Specifically, to reflect the overall performance across subjects, let ${\overline{{\rm{MAE}}}}_{k,c}$ and ${\bar{\rho }}_{k,c}$ denote the average MAE and PCC for a given ROI *k* under illumination condition *c*. The normalized score for MAE (*NS*_MAE_) was inversely scaled to assign the lowest error a score of 1, while the normalized score for PCC (*NS*_PCC_) was computed to assign the highest correlation a score of 1. The normalized scores are mathematically defined as:

3 $$N{S}_{{\rm{PCC}}}(k,c)=\frac{{\bar{\rho }}_{k,c}-{\min }_{i}({\bar{\rho }}_{i,c})}{{\max }_{i}({\bar{\rho }}_{i,c})-{\min }_{i}({\bar{\rho }}_{i,c})},$$

4 $$N{S}_{{\rm{MAE}}}(k,c)=\frac{{\max }_{i}({\overline{{\rm{MAE}}}}_{i,c})-{\overline{{\rm{MAE}}}}_{k,c}}{{\max }_{i}({\overline{{\rm{MAE}}}}_{i,c})-{\min }_{i}({\overline{{\rm{MAE}}}}_{i,c})},$$

where ${\min }_{i}(\cdot )$ and ${\max }_{i}(\cdot )$ represent the minimum and maximum average metric values obtained across all evaluated facial ROIs (*i*) under the same illumination condition *c*. Consequently, higher normalized scores consistently represent better measurement performance in that specific environment.

### Ethical considerations

The human face images displayed in Figs. 1, 5 are sample frames of subject 47 from the UBFC-rPPG dataset^53^, who has consented to publish his images in ppt, website, report, and scientific articles based on the dataset consent record. The UBFC-rPPG dataset can be accessed at https://sites.google.com/view/ybenezeth/ubfcrppg/. No identifiable personal information beyond the content provided by the public dataset was used. No artificial synthesis was utilized to process the images.

## Supplementary information

Supplementary information

## Data Availability

The datasets used in this study are available from their respective sources. The BUAA-MIHR dataset is under the Creative Commons Attribution Non Commercial Share Alike 4.0 International License (CC-BY-NC-SA-4.0): https://creativecommons.org/licenses/by-nc-sa/4.0/. The MMPD dataset requires permission from the dataset owners. The BUAA-MIHR dataset can be accessed at https://xilin1991.github.io/Large-scale-Multi-illumination-HR-Database/. The MMPD dataset can be accessed at https://github.com/McJackTang/MMPD_rPPG_dataset. All datasets were obtained through standard application procedures and used in accordance with the associated data usage agreements.

## Code availability

All code used for the experiments is open source with no restrictions at https://github.com/shuoli-roy/optimal_roi_rppg_illumination. The implementations of rPPG-related utilities are sourced from the pyVHR package^50^, which is available at https://github.com/phuselab/pyVHR.
