# Supplementary material for: Lighting effects on optimal facial regions for remote heart rate measurement
Source: NPJ Cardiovasc Health. 2026 Jun 23;3:40. doi: 10.1038/s44325-026-00140-7 (PMC13328524; doi:10.1038/s44325-026-00140-7)
Supplement: Supplementary file 1 — Supplementary information [file 44325_2026_140_MOESM1_ESM.pdf]

Supplementary information

Supplementary Note 1

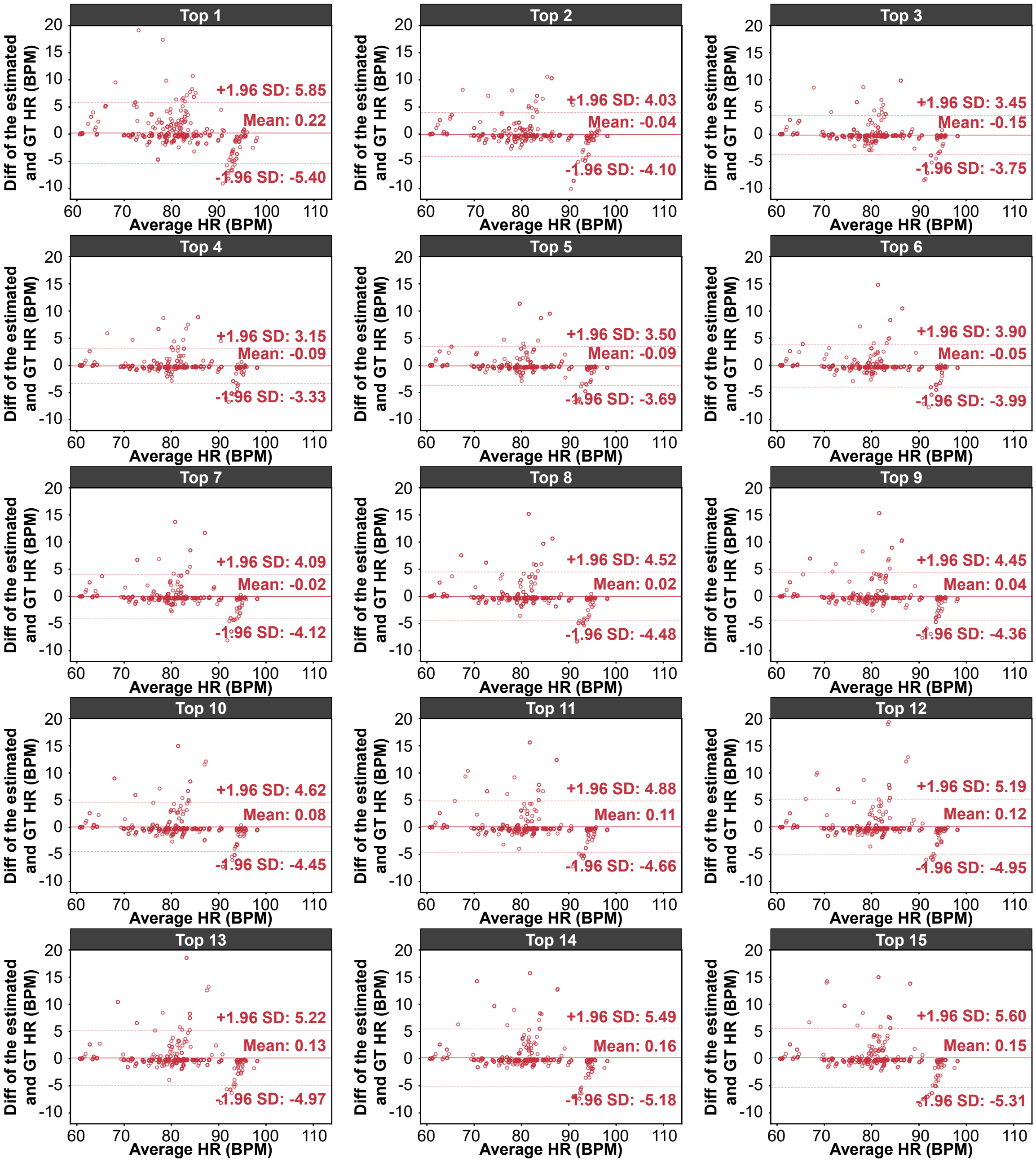

Figure S1. Bland–Altman analyses of rPPG-based HR measurement acceptance rates with gradual facial ROI expansion on the BUAA-MIHR dataset[1].

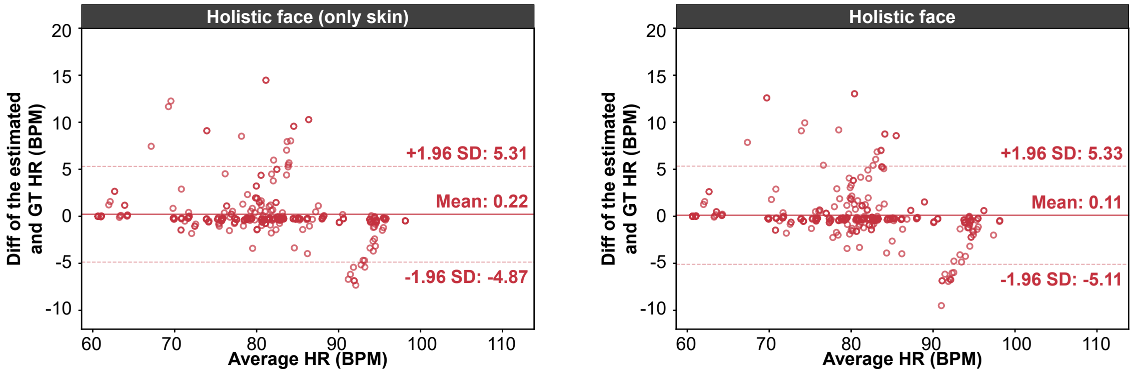

Figure S2. Bland–Altman analyses of rPPG-based HR measurement acceptance rates with holistic-face strategies on the BUAA-MIHR dataset[1].

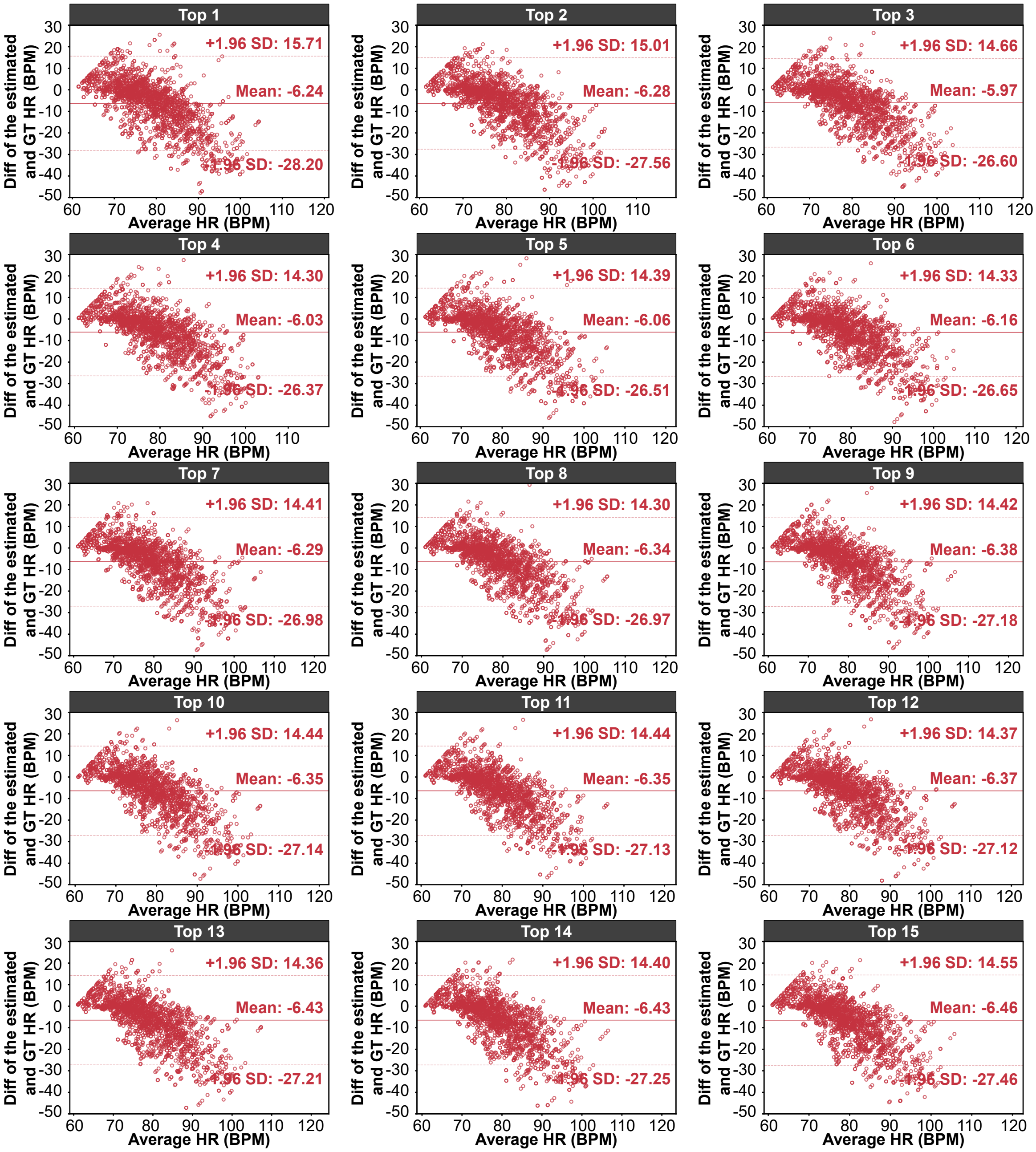

Figure S3. Bland–Altman analyses of rPPG-based HR measurement acceptance rates with gradual facial ROI expansion on the MMPD dataset[2].

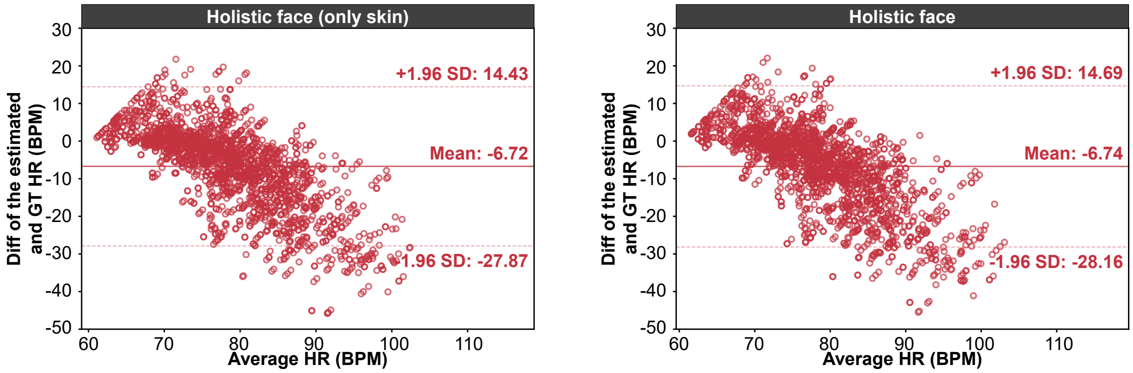

Figure S4. Bland–Altman analyses of rPPG-based HR measurement acceptance rates with holistic-face strategies on the MMPD dataset[2].

Supplementary Note 2

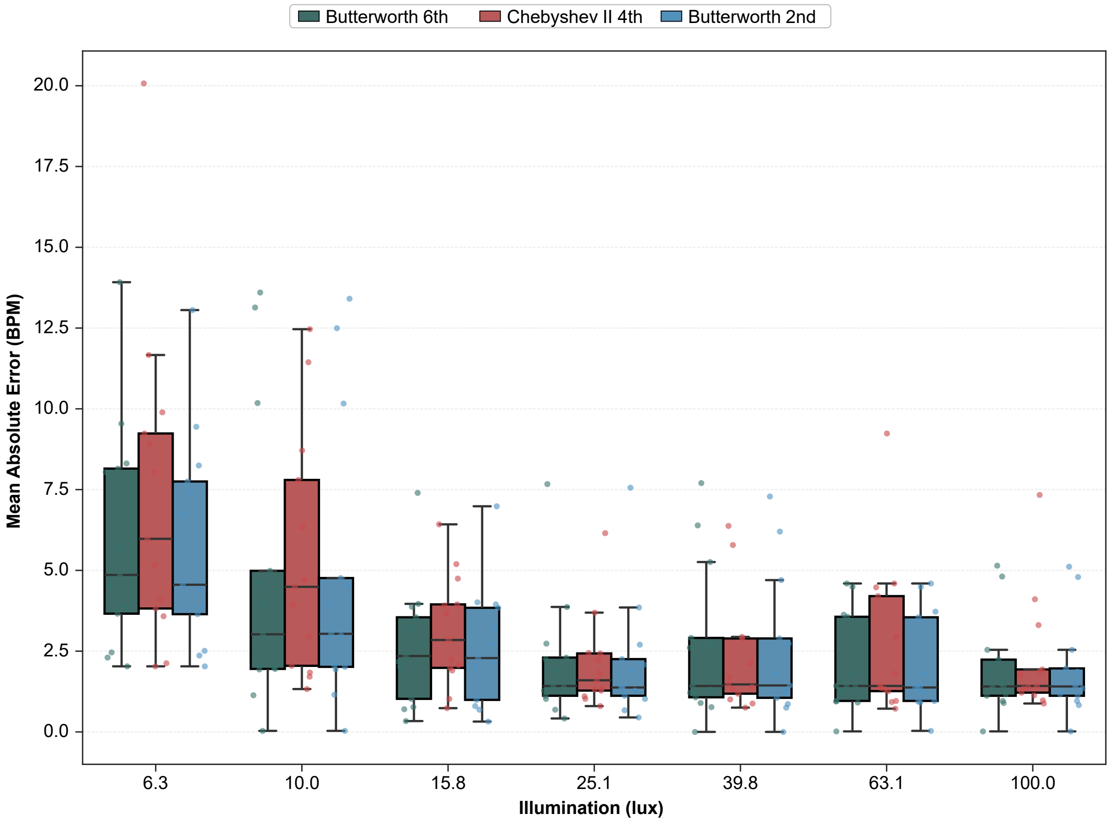

Figure S5. Comparison of filter configurations on the BUAA-MIHR dataset[1]. Mean absolute error (MAE) distributions for the glabella ROI across seven illumination levels (6.3–100.0 lux) using three filter types: a 6th-order Butterworth bandpass filter, a 4th-order Chebyshev Type II filter, and a 2nd-order Butterworth filter.

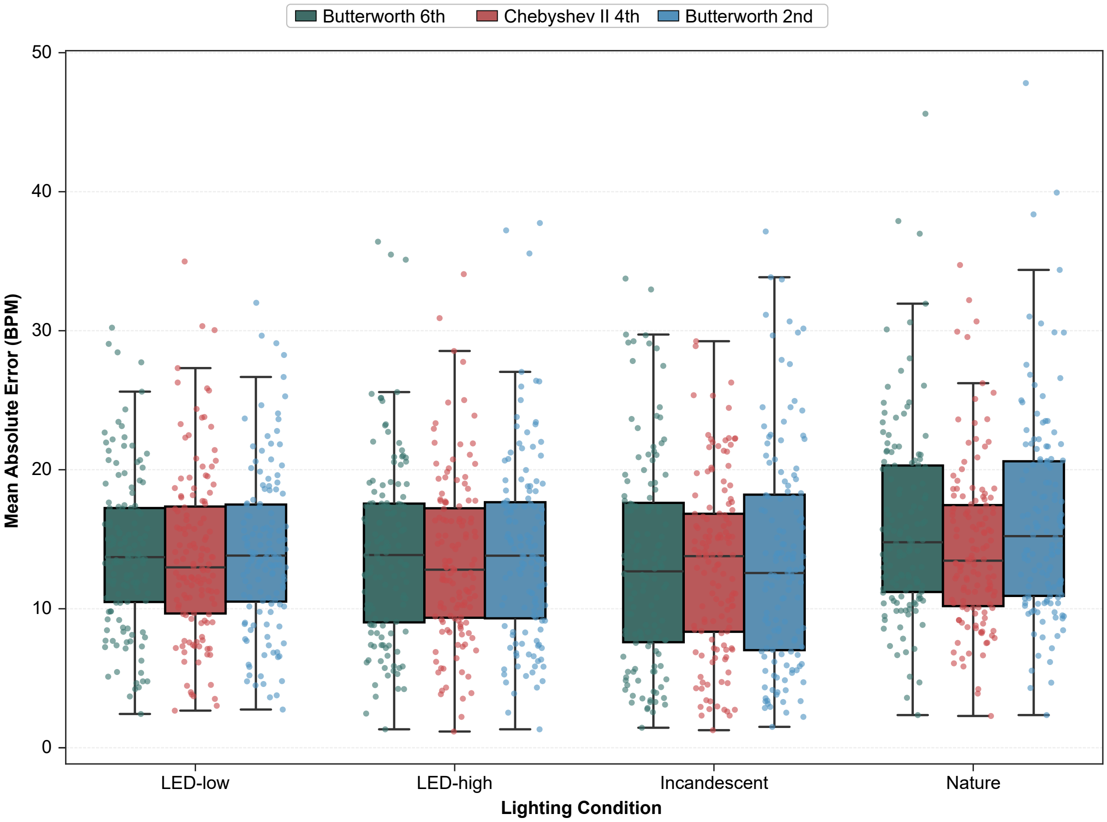

Figure S6. Comparison of filter configurations on the MMPD dataset[2]. Mean absolute error (MAE) distributions for the glabella ROI under four lighting conditions (LED-low, LED-high, incandescent, nature) using three filter types: a 6th-order Butterworth bandpass filter, a 4th-order Chebyshev Type II filter, and a 2nd-order Butterworth filter.

References

1. Xi, L., Chen, W., Zhao, C., Wu, X. & Wang, J. Image enhancement for remote photoplethysmography in a low-light environment. In *2020 15th IEEE International Conference on Automatic Face and Gesture Recognition (FG 2020)*, 1–7 (IEEE, 2020).
2. Tang, J. *et al.* MMPD: Multi-domain mobile video physiology dataset. In *2023 45th Annual International Conference of the IEEE Engineering in Medicine & Biology Society (EMBC)*, 1–5 (IEEE, 2023).
